# AGS cell line xenograft tumor as a suitable gastric adenocarcinoma model: growth kinetic characterization and immunohistochemistry analysis

**DOI:** 10.22038/IJBMS.2018.22938.5835

**Published:** 2018-07

**Authors:** Tahereh Barati, Mahnaz Haddadi, Fatemeh Sadeghi, Samad Muhammadnejad, Ahad Muhammadnejad, Ronak Heidarian, Motahareh Arjomandnejad, Saeid Amanpour

**Affiliations:** 1 Cancer Biology Research Center, Tehran University of Medical Sciences, Tehran, Iran; 2 Research Center for Molecular and Cellular Imaging, Tehran University of Medical Sciences, Tehran, Iran; 3 Vali-e-Asr Reproductive Health Research Center, Tehran University of Medical Sciences, Tehran, Iran

**Keywords:** AGS, Athymic nude mice, Immunohistochemistry, Stomach neoplasm, Xenograft model

## Abstract

**Objective(s):**

Gastric cancer is the third leading cause of cancer-related death worldwide. The overall survival rate of patients is poor because gastric cancers are usually diagnosed at the late stages. Therefore, further research is needed and appropriate research tools are required to develop novel therapeutic approaches.

**Materials and Methods:**

Eight female athymic nude mice with a *C57BL/6* background were used in this study. AGS cells were inoculated into the flank. The tumor volumes were calculated and growth curves were drawn. When the volume of the tumors reached 1000 mm3, the animals were humanely euthanized with CO_2_ gas. After harvesting, tumors were analyzed with Hematoxylin and Eosin (H&E) and immunohistochemistry (IHC). A pathologist confirmed tumor entity through H&E staining. Tumors were evaluated for expression of HER-2, P53, Ki-67, CD34, cytokeratin 8 (CK8), vimentin, estrogen receptor (ER), and progesterone receptor (PR) utilizing immunohistochemistry.

**Results:**

The tumor take rate was 62.5%, mean doubling time was 40.984 d, and the latency period was 30.62 days. The H&E staining results showed highly malignant hyperchromatin epithelial cells. IHC assessment showed the mutation status of P53 gene. The expression score of the CK8 protein in the tumor cells was +3. Vimentin protein was not expressed and changes in mesenchymal phenotype were not observed. Ki-67 IHC indicated that the proliferation rate was >43% and angiogenesis was defined as high MVD.

**Conclusion:**

The respective AGS xenograft model provides an opportunity to understand the pattern of tumor growth as well as to evaluate new gastric cancer therapies in *in vivo* studies.

## Introduction

Gastric cancer is the fourth most common malignant tumor in the world and the incidence rate is higher in the developing countries compared to the developed countries (1). Gastric cancer patients are usually diagnosed at the late stages (2). Combinational chemotherapy, radiotherapy, and surgery are used to treat patients with different stages of gastric cancer; however, the 5-year survival rate is low (3). Further research is needed and appropriate research tools are required to develop novel therapeutic approaches.

Animal models are the most practical tools in cancer research (4). Experience shows that the more appropriate disease models lead to developing better therapeutic approaches*. *Recently, several animal models for gastric cancer have been suggested, including the xenograft models (5). Tumor xenograft models established in immunodeficient mice are good cancer models for oncological studies and there is agreement that the use of these models for the development of anti-cancer drugs in the preclinical phase is beneficial. Immunodeficient animals such as athymic nude mice are inoculated with human neoplastic cells to establish such models (6).

To date, several models of gastric carcinoma xenografts using different cell lines have been developed. AGS is one of the most common cell lines for the xenograft modeling of gastric cancer; however, there is limited information about the characteristics of this model. The present study characterized the growth kinetics and cellular markers of AGS heterotopic xenograft tumors in athymic nude mice.

## Materials and Methods

*Cell lines*

All chemicals were obtained from BD Biosciences (Franklin Lake, NJ, USA). The AGS cell line was purchased from the National Cell Bank of Iran at Pasteur Institute (Tehran) and cultured in DMEM containing 10% fetal bovine serum (FBS). When the cells reached 80–90% confluency, a total of 5 × 10 ^6^ cells in 200 μl of serum-free medium was prepared for inoculation. 

***Animal***

In this laboratory experiment, 8 female 6-week-old athymic nude mice with a *C57BL/6 *genetic background were purchased from Omid Institute for Advanced Biomodels (Iran) and kept in ventilated cages individually. The animals were supplemented with autoclaved food and water *ad libitum*. This experiment was performed in accordance with the ethical principles of reduction, refinement, and replacement (7).

***Tumor implantation***

A total of 5 × 10 ^6^ cells were inoculated bilaterally and subcutaneously into the flank of the animals. It should be noted that the inoculation site should be disinfected before injection.

***Evaluation of tumor growth***

We measured tumor growth twice a week for 45 days after the tumor specimen was measurable. We calculated the volume of tumors using the standard formula (length × width^2^ × 0.52) and drew the growth curves (8). When the tumor volume reached 1000 mm^3^, the animals were humanely euthanized with CO_2_ gas.

***Immunohistochemistry***

The harvested tumor specimens were fixed in 10% buffered formaldehyde, dehydrated, embedded in paraffin wax, and stained by H&E. A pathologist confirmed the existence of the tumor entity by H&E staining. Immunohistochemistry (IHC) was then carried out using HER-2, P53, Ki-67, CD34, cytokeratin 8 (CK8), vimentin, estrogen receptor (ER), and progesterone receptor (PR) antibodies. All antibodies were purchased from Dako (Glostrup, Denmark). 

The paraffin-embedded blocks were cooled in an ice-water mixture for 30 min and 4 µm thick sections were cut and placed on slides. After brief drying, the sections were heat-fixed, deparaffinized, and rehydrated in graded ethanol. Antigen retrieval was carried out using heat and citrate buffer and immersed in 3% H_2_O_2_ and pure methanol for 5 min. The slides were washed with distilled water, incubated with the primary antibody (with negative control) for 10 min and again washed three times. The slides were incubated with secondary antibody (biotinylated link) for 10 min and washed by buffered saline for three times. IHC staining was performed by immersing the slides in diaminobenzidine as a substrate-chromogen for 10 min. 

Microscopic evaluation of HER-2 was performed on the basis of membrane immunoreactivity and scored from 0 to 3 based on the immunoreactivity rate in the malignant core cells. We did microvessel density (MVD) analysis for angiogenesis evaluation. The densities were ranked as 0 to 20 (low), 20 to 40 (moderate), or >40 (high).

All other vital organs were examined microscopically for metastatic deposits.

## Results

***Evaluation of tumor growth***

**Figure 1:**
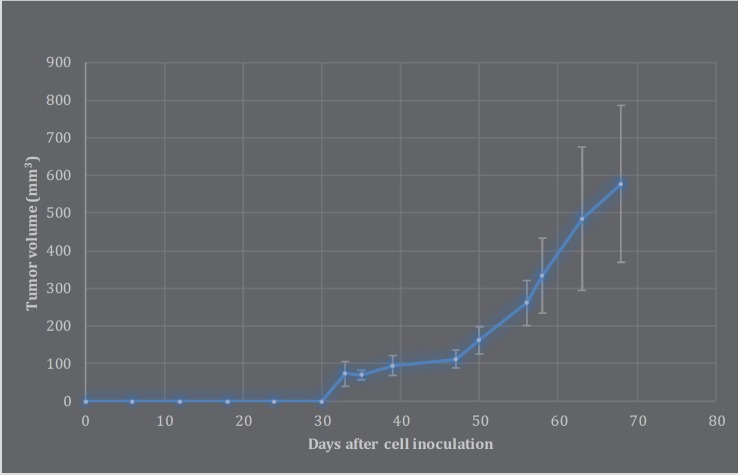
Growth curve of gastric tumors during 100 days after inoculation of AGS cells. Error bars represent standard deviation

The measurable tumors were not observed until 33 days after inoculation of AGC cells.  Subsequently, the gastric tumor volumes were measured for 45 days (Figure 1). The tumor take rate was 62.5%, mean doubling time was 40.984 days, and the latency period was 30.62 days (Figure 1). 

***Immunohistochemistry***

**Figure 2:**
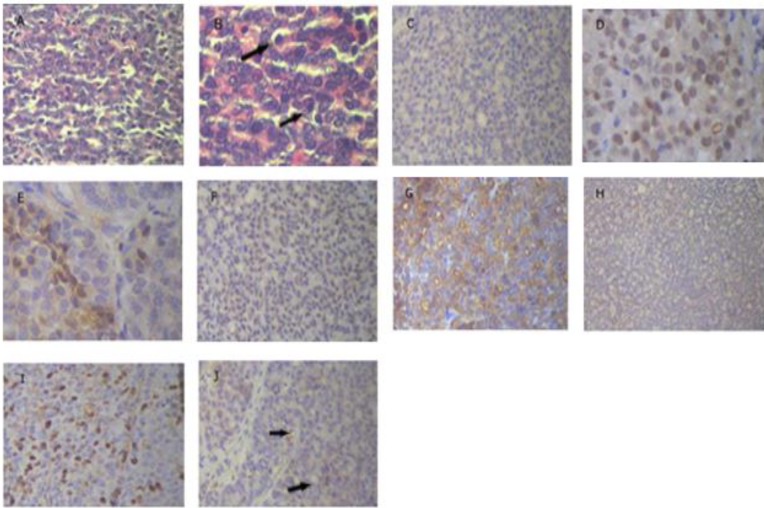
Micrographs prepared by xenograft tumor in gastric adenocarcinoma model using H&E and IHC staining. A, B) H&E staining illustrates that the tumor was a poorly differentiated adenocarcinoma. Black arrow in B shows severe polymorphism and active nucleus. C) IHC staining via HER-2 marker. There was no membrane activity immunity and score was zero. D) IHC staining via P53 marker. Nucleus was immunoreactive and indicated a mutation in the P53 tumor gene. E, F) ER & PR staining. E is related to immunoreactive, but F shows that PR marker was completely negative. G) IHC staining by CK marker, which represents tumor with epithelial origin. H) Staining via vimentin marker, which illustrates tumor not only consisted of mesenchymal tissue but in this model, EMT did not occur. I) Staining via ki-67 marker. The nucleus was immunoreactive and indicated an active cell cycle. J) IHC staining via CD34 marker. Black arrow shows microvessels

We found a poorly differentiated adenocarcinoma tumor in our AGS xenograft model (Figures 2A and 2B). However, no evidence of involvement of other organs was found. The H&E staining results showed highly malignant hyperchromatin epithelial cells. Atypical mitosis and numerous nucleoli were also observed in the cells. HER-2 expression was negative in the tumor specimens (Figure 2C). We found a mutation in the P53 gene by IHC assessment (Figure 2D). The ER-positivity rate was 8% (Figure 2E); however, PR expression was negative in the tumor (Figure 2F). The percentage of positive nucleoli was 59%. CK8 protein expression in the tumor cells was +3, which suggests the presence of intracellular filaments in support for epithelial cells (Figure 2G). Vimentin protein was not expressed and a change in mesenchymal phenotype was not observed (Figure 2H). Ki-67 IHC indicated that the proliferation rate was >43% (Figure 2I) and angiogenesis was defined as high MVD (Figure 2J).

## Discussion

The present study characterized the growth properties and cellular markers of AGS heterotopic xenograft tumor in athymic mice. A poorly differentiated adenocarcinoma was successfully established from the AGS cell line with an acceptable take rate. This xenograft model was positive for ER, P53, and CK8, but HER-2 and vimentin expressions were negative. The proliferation rate and angiogenesis were remarkable in this model. 

Tools have been developed for the study of cell biology and development of new therapeutic modalities. Park *et al.* (9) and Sekiguchi and Suzuki (10) reported a modest total of 37 gastric cancer cell lines and Park *et al.* (11) described 8 established gastric carcinoma cell lines. The first successful heterotransplantation of human tumor tissue into immunodeficient mice was reported nearly 40 years ago when Rygaard and Poulsen implanted a small piece of colon adenocarcinoma into nude mice (12). Subsequently, a xenograft model using the AGS cell line was established and AGS cells were inoculated into female athymic C57BL/6 nude mice. The latency period after inoculation of the cultured cell suspension was almost 5 weeks after injection. 

Young-Kwang Yoon *et al.* investigated signaling pathways to create an AGS xenograft model of nude mice (13). Changhua Zhang assessed peritoneal dissemination of gastric cancer and concluded that peritoneal gastric cancer xenografts were successful after intraperitoneal injection of NCI-N87 and SNU16 cells (14). The orthotopic nude mouse model for preclinical research of gastric cardia cancer was established by Bhargava (15). Immunohistochemical array analysis results showed positivity for CK and negativity for vimentin. In this model, epithelial properties were maintained and epithelial-mesenchymal transition (EMT) did not occur.

The epithelial filaments providing support for EMT play an important role in the formation of a body plan and in the differentiation of tissues and organs. EMT is partly responsible for tissue repair but can cause organ fibrosis and promote the progression of cancer through a variety of mechanisms. EMT gives cells migratory and invasive properties, influences stem cell properties, and causes apoptosis and senescence. It also aids immunosuppression. The mesenchymal state is connected with the capacity of cells to migrate to distant organs and to stemness. It also allows their subsequent differentiation into multiple cells and metastasis (16).

The HER-2 was negative; this was likely because 25% to 30% of human gastric adenocarcinomas are considered to be HER-2 negative (17). Although evaluation of ER and PR is necessary for breast cancer, it can also form part of a therapeutic strategy in other cancers. In the present study, ER was negative, but it was essential to evaluate ER to enhance the evidence-based data. Mutation in the P53 gene decreases the survival rate in patients with gastric cancer (18). In xenograft models using this type of cell line, the P53 is positive, which provides an opportunity for researchers to evaluate targeted therapy against P53 and to control this gene mutation in gastric cancer through gene therapy methods. 

The increase observed in Ki-67 in this model can help researchers control the cell cycle, and detection of a high rate of angiogenesis provides the opportunity to control angiogenesis in gastric cancer. At present, control of angiogenesis is a goal in the treatment of gastric cancer. We hope to identify a gene profile for gastric cancer and determine new therapeutic strategies.

One limitation of this study was the lack of data from a xenograft model. Although this model has been used frequently, characterization of AGS xenograft tumors using various molecular markers and evaluation of angiogenesis requires further work. The lack of data from xenograft models is another limitation. Further investigation is required to characterize this model using an AGS cell line. It is hoped that the development of a comprehensive model of AGS xenografts for *in vivo* investigation can enable researchers to investigate new treatments for gastric cancer.

## Conclusion

The respective AGS xenograft model provides an opportunity to understand the pattern of tumor growth as well as to evaluate new gastric cancer therapies in *in vivo* studies.
